# Low-grade myxofibrosarcoma following a metal implantation in femur: a case report

**DOI:** 10.1186/1746-1596-9-6

**Published:** 2014-01-20

**Authors:** Weisong Li, Dan Li, Xiansen Zhu, Shaohui Lu, Chunlei He, Qingchun Yang

**Affiliations:** 1 Department of Pathology, Gannan Medical University, No. 1, Yixueyuan Road, Ganzhou, Jiangxi 341000, China; 2 Department of Pathology, the First Affiliated Hospital, Sun Yat-Sen University, No. 58, Zhongshan Road II, Guangzhou, Guangdong 510080, China; 3 Translational Medicine Research and Development Center, Shenzhen Institutes of Advanced Technology, Chinese Academy of Sciences, No. 1068 Xueyuan Avenue, Shenzhen, Guangdong 518055, China; 4 Department of Radiology, the First Affiliated Hospital, Gannan Medical University, No. 23, Qingnian Road, Ganzhou, Jiangxi 341000, China; 5 Department of Orthopedics, the First Affiliated Hospital, Gannan Medical University, No. 23, Qingnian Road, Ganzhou, Jiangxi 341000, China

**Keywords:** Myxofibrosarcoma, Femur, Fracture, Titanium alloy, Metal implantation

## Abstract

Myxofibrosarcoma is a myxoid variant of malignant fibrous histiocytoma that most commonly involves the extremities of elderly people. However, a primary myxofibrosarcoma with bone invasion in young adults is extremely rare. Herein, we report the case of a 31-year-old male with a gradually enlarging left thigh mass, who had a history of left femur fracture and received an open reduction and internal fixation with titanium alloy plates and screws 33 months previously. Imaging investigations revealed an irregularly shaped soft tissue mass around the left femur shaft and a partial bone defect in the middle one-third of the left femur. Pathological examination of the resected specimen showed a multi-nodular appearance, abundant myxoid matrix and elongated curvilinear capillaries. Immunohistochemical studies revealed that the tumor cells was positive for VIM and MDM2, and was negative for CK, MSA, SMA, DES, S-100 and CD34. Labeling index of Ki-67 was 25%. Based on the morphological finding and immunostaining, it was diagnosed as a low-grade myxofibrosarcoma. The clinical and imaging examinations did not reveal the evidence of a primary cancer elsewhere, and the patient had no personal or family history of malignancy. To our knowledge, this is the first case of a primary myxofibrosarcoma developed following a fracture and metal implantation in young adults.

**Virtual slides:**

The virtual slide(s) for this article can be found here: http://www.diagnosticpathology.diagnomx.eu/vs/1745984882113605

## Background

Myxofibrosarcoma is a myxoid variant of malignant fibrous histiocytoma (MFH), characterized by a nodular appearance, prominent myxoid matrix, elongated curvilinear capillaries, and location mostly in dermal and subcutaneous tissues in the extremities of elderly people [1-3]. In 1996, Mentzel et al. [3] reported 75 cases of myxofibrosarcomas and divided these tumors into low-, intermediate-, and high-grade categories, depending on the degree of cytologic atypia and the presence or absence of pleomorphic MFH-like lesion within the tumor. Tumors of the low-grade category showed a mainly myxoid with mild cytologic atypia, while high-grade tumors showed a pleomorphic appearance with multinucleated giant cells, high mitotic activity, and areas of necrosis.

Several clinical studies of myxofibrosarcomas showed that approximately 80% of the tumors occurred in the extremities and about 12% in the trunk, with a high incidence in the dermal or subcutaneous tissues [1-3]. Other sites for myxofibrosarcomas included head and neck region, retroperitoneum, pelvis and heart [3,4]. However, a primary myxofibrosarcoma with bone invasion was rarely reported [5-11]. In addition, the peak age incidence of myxofibrosarcomas was in the fifth to seventh decades with a slight male predominance [2,12].

Herein, we report a low-grade myxofibrosarcoma with left femur invasion in a 31-year-old male, and present the clinical, radiological and histopathological characteristics of this tumor. The clinical and imaging examinations did not reveal the evidence of a primary cancer elsewhere, and the patient had no personal or family history of malignancy. It is noteworthy that the patient had a history of left femur fracture and received an open reduction and internal fixation with titanium alloy plates and screws 33 months previously. To our knowledge, this is the first report on a primary myxofibrosarcoma that developed following a fracture and metal implantation in young adults.

## Case presentation

A 31-year-old male patient has a history of a multiple fracture involving the left femur shaft, the middle and distal segment of the left tibiofibula, as a result of a traffic accident in October 2008. Subsequently, the patient underwent an open reduction and internal fixation (ORIF), using titanium alloy plates and screws. Ten months after ORIF, an X-ray examination showed no displacement of the fracture site, and the bridging callus was visible on two standard views with partial obliteration of the fracture line. However, the patient did not undergo reoperation to remove the metal implants due to financial difficulties.

In August 2011, the patient complained of a 3-month history of a gradually enlarging left thigh mass. Physical examination revealed a 12 × 10 cm firm, tender and fixed mass in the anteromedial left thigh, with local superficial venous engorgement and skin temperature increment. Percussion pain in the axial direction of the left lower limb was absent. The inguinal lymph nodes were not palpable. Routine chest radiograph and abdominal ultrasound examination showed no abnormalities. Other routine tests, including plasma biochemistry, liver function tests and urinalysis, were normal.

**Figure 1:**
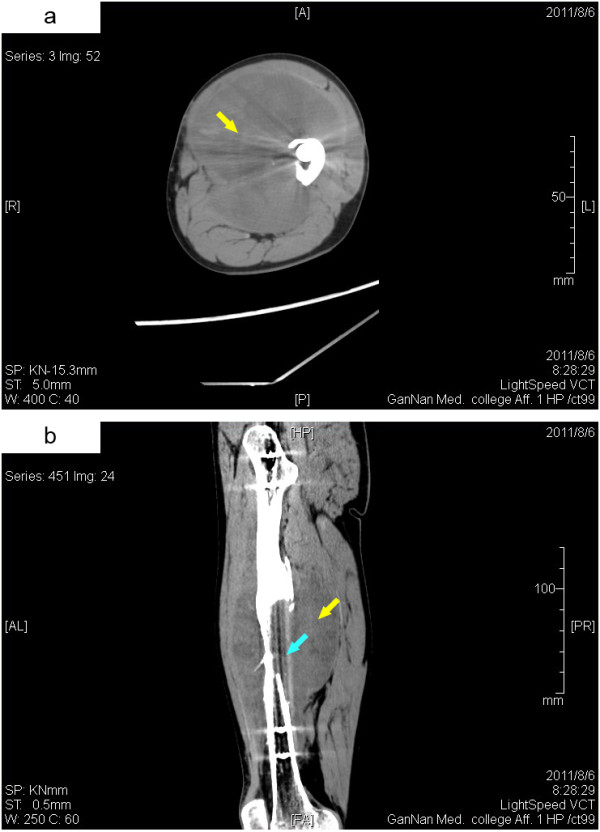
**CT images of the left thigh. (a)** Transverse image. **(b)** Coronal image. A high-resolution volumetric CT scanning revealed a 9.7 × 11 × 19 cm irregularly shaped soft tissue mass in the anteromedial left thigh (yellow arrow) and a partial bone defect approximately 9.7 cm in length on the medial border of the left femur shaft (light blue arrow).

The X-ray examination demonstrated an ill-defined osteolytic lesion with disappearance of the medial cortical bone in the middle of the left femur shaft, as well as an ambiguous image of a soft tissue mass. Computed tomography (CT) images clearly showed a partial bone defect approximately 9.7 cm in length on the medial border of the left femur shaft. A 9.7 × 11 × 19 cm irregularly shaped soft tissue mass was observed around the left femur, with the surrounding tissues compressed and shifted significantly (Figure 1). CT findings also showed that the soft tissue mass was heterogeneous in density with CT value of 36 HU, in which small pieces of slightly high-density lesions were found. These imaging findings suggested that the mass was neoplastic.

**Figure 2:**
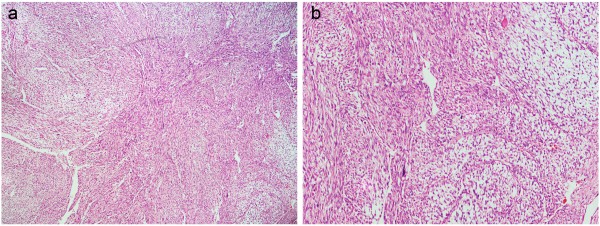
**Histopathological examination of the tumor. (a)** Magnification, ×40. **(b)** Magnification, ×100. H&E staining showed that the tumor was predominantly composed of a large number of diffuse fusiform cells and myxoid matrix. The fusiform tumor cells were arranged in a multi-nodular pattern, with indistinct cell margins, slightly eosinophilic cytoplasm, and hyperchromatic atypical nuclei. Mitoses were infrequent. Many elongated curvilinear capillaries were observed.

Tissue biopsy was performed, and histopathological examination reported that the tumor was predominantly composed of a large number of diffuse fusiform cells and myxoid matrix. The fusiform tumor cells were arranged in a multi-nodular pattern, with indistinct cell margins, slightly eosinophilic cytoplasm, and hyperchromatic atypical nuclei. Mitoses were infrequent. In addition, many elongated curvilinear capillaries were observed (Figure 2). These cytological findings suggested a malignant tumor of mesenchymal origin.

On 3 September 2011, the patient underwent operation for the metal implants removal and tumor resection. Intraoperative findings indicated that the tumor infiltrated into the middle of the femur shaft and the surrounding tissues including the rectus femoris, sartorius, pectineal muscle, long adductor muscle, gracilis muscle, vastus medialis, vastus lateralis, vastus intermedius, and biceps femoris. The tumor and bone tumor segment approximately 15 cm in length were resected, and the surgical margin was microscopically free of tumor. Sequential bone defect reconstruction after tumor resection was performed by reimplantation with the alcohol inactivated bone tumor segment, in which the vascularised autologous left fibula approximately 18 cm in length was inserted. Then, an internal fixation with titanium alloy plates and screws was once again performed, and a closed vacuum drainage system was used.

**Figure 3:**
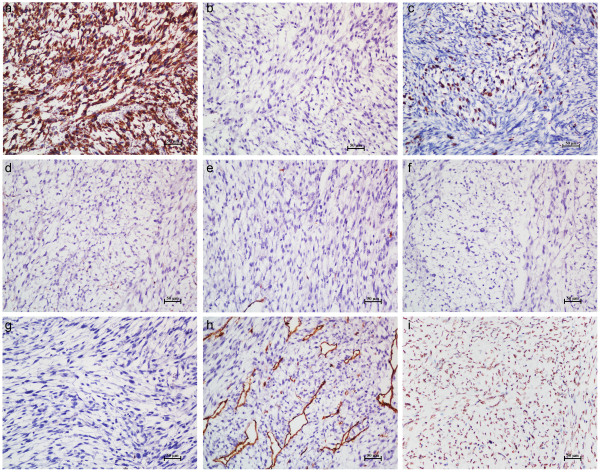
**Immunohistochemical studies of the tumor. (a-i)** Magnification, ×200. The tumor cells expressed strong immunoreactivity for VIM **(a)** and negative for CK **(b)**. Labeling index of Ki-67 **(c)** was 25%. MSA **(d)**, SMA **(e)**, DES **(f)** and S-100 protein **(g)** were negative in the tumor cells. **(h)** CD34 staining was detected in vascular endothelial cells rather than in tumor cells, highlighting the curvilinear capillaries. **(i)** MDM2 was positive in the tumor cells.

Macroscopically, the excised specimen was a grayish-white or grayish-brown mass measured 7 × 14 × 10 cm. On the cut section, a fish-like appearance with central necrosis area was observed. Microscopic features were identical to that of the biopsy. Immunohistochemical studies showed that the tumor cells expressed strong immunoreactivity for vimentin (VIM) and negative for cytokeratin (CK), suggesting a mesenchymal histogenesis (Figure 3a and b). Labeling index of Ki-67 (the number of Ki-67 positive tumor cells divided by the sum of Ki-67 positive and negative tumor cells) was 25% (Figure 3c). Muscle-specific actin (MSA), smooth muscle actin (SMA) and desmin (DES) were negative in the tumor cells, eliminating the possibility of a muscle-derived tumor (Figure 3d-f) [13]. The negative expression of S-100 protein eliminated a tumor of neural or adipose tissue origin (Figure 3g). The tumor cells were negative for cluster of differentiation 34 (CD34) (Figure 3h), B cell lymphoma/lewkmia-2 and anaplastic lymphoma kinase, which eliminated the possibility of a solitary fibrous tumor or inflammatory myofibroblastic tumor [14]. Interestingly, the positive staining of CD34 in vascular endothelial cells highlighted the curvilinear capillaries, which was an important histological feature of myxofibrosarcoma. In addition, positive expression of murine double minute 2 (MDM2) was observed in the tumor cells. Although MDM2 immunostaining is useful adjunct in diagnosing well-differentiated and dedifferentiated liposarcoma subtypes, overexpression of MDM2 has also been reported in a small number of myxofibrosarcomas [15-17]. The combination of clinical and pathological features revealed a low-grade myxofibrosarcoma.

The patient received three cycles of postoperative adjuvant chemotherapy with a combination of ifosfamide, doxorubicin, cisplatin and mesna, and he experienced no special discomfort during chemotherapy. In June 2012, the patient underwent the third operation for the removal of metal implants due to implant breakage in an accidental fall, followed by external fixation.

**Figure 4:**
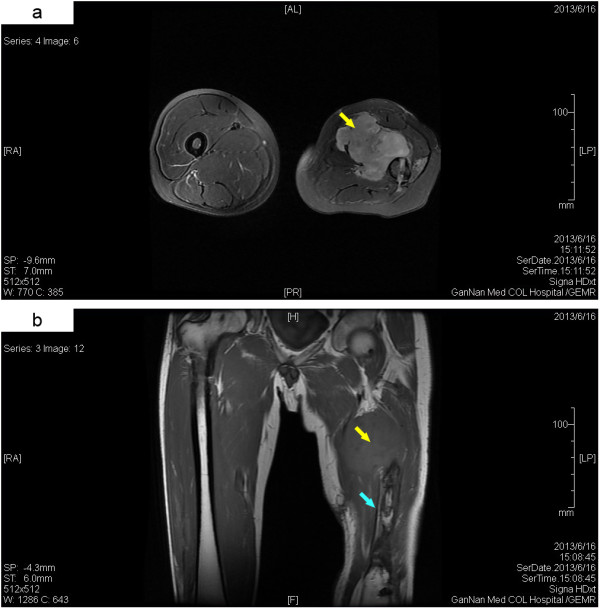
**MRI images of the thigh. (a)** Transverse T2-weighted image. **(b)** Coronal T1-weighted image. MRI images showed an irregular soft tissue mass with ill-defined margin in the anteromedial left thigh (yellow arrow), accompanied by surrounding soft tissue swelling and normal muscle structure disappearance. Bone destruction in the left femur shaft was observed (light blue arrow).

**Figure 5:**
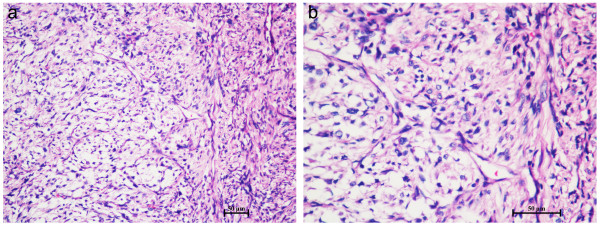
**Histopathological examination of the recurrent tumor. (a)** Magnification, ×200. **(b)** Magnification, ×400. H&E staining showed that the tumor cells exhibited increased atypia and pleomorphism compared with those in the primary tumor, varying from small and bland to enlarged, bizarre, pleomorphic and multinucleated. Abundant myxoid matrix and elongated curvilinear capillaries were also observed.

However, in June 2013, only 22 months after tumor resection, a firm and painless mass approximately 5 cm in diameter was palpated in the medial left thigh. Magnetic resonance imaging (MRI) showed an irregular soft tissue mass with ill-defined margin in the anteromedial left thigh, accompanied by surrounding soft tissue swelling and normal muscle structure disappearance. Additionally, bone destruction in the left femur shaft was also observed (Figure 4). Tissue biopsy was performed and histopathological examination showed that the tumor exhibited the classical histological features of myxofibrosarcoma, including a nodular appearance, prominent myxoid matrix and elongated curvilinear capillaries. The morphology of the tumor cells varied from small and bland to enlarged, bizarre, pleomorphic and multinucleated, suggesting increased atypia and pleomorphism compared with those in the primary tumor (Figure 5). The combination of clinical and pathological features revealed a recurrent myxofibrosarcoma with higher malignancy. There was no evidence of distant metastasis. The patient eventually underwent left hip disarticulation due to the inability to control the tumor progression.

## Discussion

Myxofibrosarcoma, also known as a myxoid variant of MFH, is one of the most common sarcomas in the extremities of elderly people, characterized by a high frequency of local recurrence. Distinctive histological features included the following: a commonly nodular growth pattern; a myxoid matrix containing elongated, curvilinear capillaries; and fusiform, round or stellate tumor cells with indistinct cell margins, slightly eosinophilic cytoplasm, and hyperchromatic atypical nuclei [3]. These lesions varied from a hypocellular, mainly myxoid, and purely spindle-cell appearance (low-grade neoplasms) to high-grade, pleomorphic (MFH-like) lesions with multinucleated giant cells, high mitotic activity, and areas of necrosis [3]. The most common sites for this tumor are the extremities, predominantly subcutaneous. Primary myxofibrosarcomas with bone invasion were rarely occurred, with no more than 15 previous cases worldwide being reported in the literature [5-11]. Five of these patients developed myxofibrosarcoma in a long bone of the extremities, with an average age of 53.2 years (range, 31 to 84). The clinicopathological features of them were summarized in Table 1.

**Table 1:** Clinicopathological features of the reported cases of myxofibrosarcomas in bone of the extremities

**Reference**	**Year**	**Age (y)/sex**	**Size**	**Location**	**Histology**	**Immunohistochemistry**	**Follow up (y)**
Kapur P et al. [5]	2004	31/M	5 cm in maximum dimension	Left tibia	nodular contour; myxoid stroma; spindle to stellate cells with mild cytologic atypia and rare mitoses	VIM (+), MSA (+), SMA (+), CD68 (-), Mac-387 (-), factor XIIIa (-), DES (-)	Not reported
Marotta D et al. [6]	2009	84/M	8 × 5 cm	Left tibia	spindle cells; polygonal epithelioid cells with pleomorphism and elevated mitotic index; necrosis	CD34 (-), SMA (-), DES (-), caldesmon (-), CK (-), S-100 (-)	2
Romeo S et al. [7]	2012	39/M	Not reported	Lower limbs	spindle cells; myxoid stroma; elongated curvilinear thin-walled blood vessels	CK (-), EMA (-), SMA (-), DES (-), caldesmon (-)	1.8
Romeo S et al. [7]	2012	44/F	Not reported	Lower limbs	spindle cells; myxoid stroma; elongated curvilinear thin-walled blood vessels	CK (-), EMA (-), SMA (-), DES (-), caldesmon (-)	6
Romeo S et al. [7]	2012	68/M	Not reported	Lower limbs	spindle cells; myxoid stroma; elongated curvilinear thin-walled blood vessels	CK (-), EMA (-), SMA (-), DES (-), caldesmon (-)	13

The etiology of myxofibrosarcoma remains unknown. It was previously reported that 2 patients developed a myxofibrosarcoma after radiotherapy [18,19]. In the current case, the patient developed a low-grade myxofibrosarcoma in the left thigh after a multiple fracture and metal implantation suffered about 3 years ago. The patient was a farmer with no known history of potential environmental hazardous substances exposure, and he was an occasional smoker and did not drink alcohol. Except for receiving a metal implantation for osteosynthesis, the patient had no relevant medical or family history. In addition, the patient has no clinical or radiological evidence of a neoplasm elsewhere in the body, excluding the possibility of a secondary lesion.

There were some literatures about the tumors developed following a bone fracture. URIST [20] reported a desmoid tumor developed following a simple fracture of the radius and ulna. Langer et al. [21] reported a case of a 19-year-old man who developed a giant cell tumor in the distal ulna following a scaphoid fracture. However, whether pure fracture plays a role in the genesis of primary bone tumors remains controversial, because of the lack of direct evidence. In addition, a series of literatures have been published with emphasis on the potential carcinogenic effect of metal implants. As early as 1956, Oppenheimer et al. [22] observed the carcinogenic effect of silver, tantalum, vitallium and stainless steel by subcutaneously embedding metal foils in rats. Clinical studies on the carcinogenicity of metal orthopedic implants showed that a similar tumor category, predominantly sarcomas, developed at the implant site and the latent periods for tumor development ranged from 2 years to more than 30 years [23-42]. The detailed information of these cases was listed in Table 2. There was no specific association between tumor type and metal material. In this case, the metal implants were made of titanium alloy. Adams et al. [39] reported a case of a high-grade osteosarcoma occurring at the site of a modular porous-surfaced titanium and cobalt alloy total hip prosthesis 3 years after device implantation. Nuevo-Ordóñez et al. [43] detected an increased titanium concentration in the serum of patients with different types of titanium implants. Although the local and systemic long-term effects of titanium particles on the human organism were unknown, their toxic effects were revealed in cell cultures [44]. This study showed that the particulate debris from a titanium metal prosthesis induced genomic instability, mainly consisting of chromatid breaks and reproductive failure, in primary human fibroblast cells. These delayed effects were similar to those caused by heavy metals cadmium and nickel [44].

**Table 2:** Patients with tumors at the site of metal implants

**Reference**	**Year**	**Age (y) at implant/sex**	**Implant site**	**Implant material**	**Tumor type**	**Latent period (y)**
McDougall A [23]	1956	12/M	Humerus	Stainless steel plate (FeCrNi) and screws (FeCr)	Ewing’s sarcoma	30
Delgado ER [24]	1958	37/M	Tibia	Eggers’ plate and screws	Osteosarcoma	3
Dube VE et al. [25]	1972	58/M	Tibia	Steel plate and screws	Hemangioendothelioma	26
Tayton KJ [26]	1980	4/F	Femur	Sherman plate (CoCrMo) and screws	Ewing’s sarcoma	7
McDonald I [27]	1981	31/M	Tibia	Sherman plate (CoCrMo) and screws	Histiocytic lymphoma	17
Penman HG et al. [28]	1984	75/F	Hip	Cobalt-chrome prosthesis	Osteosarcoma	16
Bagó-Granell J et al. [29]	1984	75/F	Hip	Charnley-Müller prosthesis	Malignant fibrous histiocytoma	2
Swann M [30]	1984	63/M	Hip	McKee-Farrar prosthesis (CoCrMo)	Malignant fibrous histiocytoma	3
Weber PC [31]	1986	76/F	Knee	CoCrMo prosthesis	Epitheliod sarcoma	4.5
Hughes AW et al. [32]	1987	14/M	Femur	Sherman screw (CoCr)	Malignant fibrous histiocytoma	29
Haag M et al. [33]	1989	69/F	Hip	Weber-Huggler prosthesis	Malignant fibrous histiocytoma	14
Ward JJ et al. [35]	1990	65/F	Femur	Smith-Petersen nail	Osteosarcoma	9
Khurana JS et al. [36]	1991	25/M	Femur	Hansen Street intramedullary nail	Malignant fibrous histiocytoma	14
Gatti GM et al. [37]	1999	37/M	Femur	Stainless steel implant	Rhabdomyosarcoma	11
Hinarejos P et al. [38]	2000	28/M	Tibia	Stainless steel plate and screws	Fibrosarcoma	30
Adams JE et al. [39]	2003	63/M	Hip	Titanium and cobalt alloy prosthesis	Osteosarcoma	3
Albert A et al. [40]	2009	75/F	Knee	Insall Burstein prosthesis	Angiosarcoma	10
Palraj B et al. [41]	2010	70/M	Tibia	Stainless steel plate and screws	Anaplastic large T-cell lymphoma	7
Dorrestijn O et al. [42]	2011	58/M	Femur	Unreamed intramedullary nail	Osteosarcoma	2

Case reports of malignant tumors at the site of metal implants in humans and domestic animals have implicated carcinogenesis as a potential, although rare, complication of metal orthopedic implants. The actual incidence of tumors in association with metal implants cannot be reliably estimated, considering that clinicians and veterinarians are not obliged to report adverse reactions to implants. Although the possibility that the apparent association is coincidental cannot be excluded, orthopedic surgeons should be aware of the potential carcinogenic risk of metal implants.

## Conclusion

In summary, we described a case of low-grade myxofibrosarcoma with bone invasion, which developed following a fracture and metal implantation in a young adult. To our knowledge, this case is the first report of primary myxofibrosarcoma in association with titanium alloy implants, which should alert doctors to the possible carcinogenic risk of titanium alloy implants.

## Consent

Written informed consent was obtained from the patient for publication of this Case report and any accompanying images. A copy of the written consent is available for review by the Editor of this journal.

## Abbreviations

CD34: Cluster of differentiation 34; CD68: Cluster of differentiation 68; CK: Cytokeratin; CT: Computed tomography; DES: Desmin; MDM2: Murine double minute 2; MFH: Malignant fibrous histiocytoma; MRI: Magnetic resonance imaging; MSA: Muscle-specific actin; ORIF: Open reduction and internal fixation; SMA: Smooth muscle actin; VIM: Vimentin.

## Competing interests

The authors declare that they have no competing interests.

## Authors’ contributions

WSL, DL and QCY collected the data, planned and drafted the manuscript. WSL and XSZ were the pathologists who evaluated the specimen. SHL was the radiologist who provided the radiology images. CLH was the clinician who cared for the patient and provided the clinical data. All authors read and approved the final manuscript.
